# Vitamin-mineral supplements and cognition among adults aged 65 and older: multiple cross-sectional population-based studies

**DOI:** 10.1007/s00394-025-03700-2

**Published:** 2025-05-24

**Authors:** Daniela Marques, Martin Preisig, Pedro Marques-Vidal

**Affiliations:** 1 University of Lausanne, Lausanne, Switzerland; 2 Department of Psychiatry, Lausanne University Hospital and University of Lausanne, Lausanne, Switzerland; 3 Department of Medicine, Internal Medicine, Lausanne University Hospital (CHUV) and University of Lausanne, Office BH10-642, Rue du Bugnon 46, Lausanne, 1011 Switzerland

**Keywords:** Cognition, Vitamin supplements, Elderly people, Cross-sectional study

## Abstract

**Purpose:**

Many people consume vitamin-mineral supplements (VMS), to prevent cognitive decline or enhance cognition. We assessed the association between VMS intake and cognition.

**Methods:**

Data from three follow-ups of the population-based CoLaus|PsyColaus cohort. Participants aged ≥ 65 years were included and categorized as VMS consumers or non-consumers. Cognitive tests included the Mini-Mental State Examination (MMSE), Stroop colour test, the CERAD praxis items, lexical and semantic fluency tasks, and the Grober and Buschke episodic memory test.

**Results:**

There were 925 (64.5% women), 836 (41.1%), and 516 (29.4%) participants from the first (2009–2013), second (2014–2018) and third (2019–2021) follow-ups, respectively. After multivariable adjustment, no significant differences were found between VMS non-consumers and consumers regarding almost all cognitive tests. The multivariable-adjusted mean ± SEM of MMSE for VMS non-consumers vs. consumers were 29.27 ± 0.06 vs. 29.28 ± 0.09, 29.21 ± 0.06 vs. 29.28 ± 0.07 and 29.32 ± 0.08 vs. 29.21 ± 0.09 for the first, second, and third follow-ups, all *p* > 0.05. The exceptions were Stroop C, where non-consumers had a statistically better but clinically irrelevant performance than consumers at the third follow-up: effect size 0.30 (0.01; 0.58) *p* = 0.042 and the Grober and Buschke test in the first follow-up, where VMS consumers scored better than non-consumers in free recall: 9.11 ± 0.15 vs. 8.55 ± 0.10 (*p* = 0.003), with opposite findings in cued recall: 5.99 ± 0.14 vs. 6.48 ± 0.09 (*p* = 0.004).

**Conclusion:**

We found no clinically significant association between VMS use and cognitive performance.

**Graphical Abstract:**

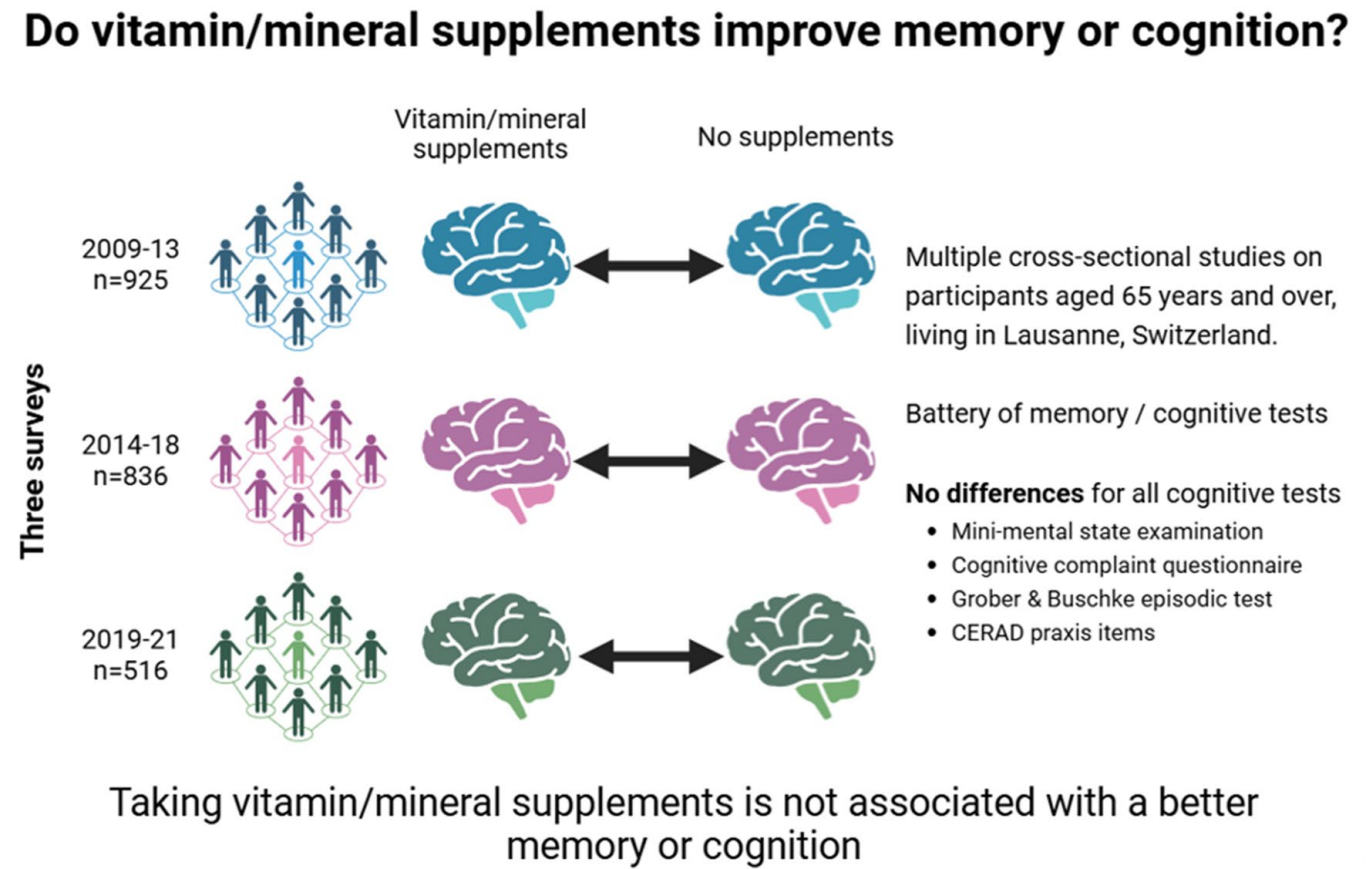

**Supplementary Information:**

The online version contains supplementary material available at 10.1007/s00394-025-03700-2.

## Introduction

Switzerland’s demographic growth follows global trends, with an increase in the proportion of the population aged 65 years or over [1]. Several meta-analyses have shown an association between age and the incidence of dementia, and it is estimated that both the incidence and prevalence of dementia will continue to increase [2].

A suggested and popular approach to slowing cognitive impairment and boosting memory is to take vitamins [3, 4]. Contrary to popular belief, there is conflicting evidence demonstrating that vitamins or food supplements provide cognitive health benefits. Randomized trials and systematic reviews have shown no advantage from vitamin D supplementation in adults [5] or in the elderly [6, 7], except some studies conducted in China [8, 9]. Negative findings were also reported when conducting mendelian randomization [10]. Additionally, a study conducted in Switzerland found no difference in the effects of various doses of vitamin D on cognitive performance in participants aged > 60 years [11]. The results of research on B vitamins have been inconsistent and inconclusive; individuals aged > 65 years with high levels of omega-3 fatty acids [12] and those aged > 50 years or older deficient in B vitamins [4, 13] have benefited the most. Multi-vitamin mixtures do not seem to have any benefit in people with mild cognitive impairment [14], or in people in cognitive healthy status [15], while a benefit has been reported in healthy elderly (≥ 65 years) [16]. Similarly, some blends with omega-3, vitamins A and E seem to improve some cognitive scores in elderly subjects, but these findings are based on a few studies with small sample sizes: 60 subjects for a study on omega-3 fatty acids, carotenoids and vitamin E [17], and 46 subjects for a study on omega-3 and omega-6 fatty acids plus antioxidant vitamins [18].

Considering those conflicting results, we aimed to assess the association between vitamin-mineral supplement (VMS) intake and cognition in a sample of apparently healthy, community-dwelling elderly (≥ 65 years) people, using data from the CoLaus|PsyColaus study.

## Methods

### Study setting

The CoLaus|PsyCoLaus study is a population-based study investigating the epidemiology and genetic determinants of psychiatric and cardiovascular disease in Lausanne, Switzerland [19]. Briefly, a representative sample was collected through a simple, non-stratified random sampling of 19,830 individuals (35% of the source population) aged between 35 and 75. Recruitment began in June 2003 and ended in May 2006. The first follow-up was performed between April 2009 and September 2013. The second follow-up was performed between May 2014 and April 2018. The third follow-up was performed between April 2019 and May 2021. As most cognitive tests were performed starting at the first follow-up, only data from the follow-ups will be used. Also, as cognitive deficiency or cognitive decline is rare in subjects aged < 65 years [20], it was decided to focus only on participants aged ≥ 65 years.

Distribution of age categories [65–70], [70–75], and [75–80] between the CoLaus|PsyCoLaus study and the population of canton Vaud where Lausanne is located showed a relatively good agreement (supplementary Table 1).

### Vitamin / dietary supplement consumption

Participants reported all medicines and vitamin-mineral or dietary supplements prescribed or bought over the counter. VMS were defined according to the Swiss compendium (compendium.ch/home/fr). If the product was not listed in the Swiss compendium, further searches on the internet were conducted. Due to wide differences in the composition of Swiss VMS [21] and to inaccurate reporting (i.e., reporting “multivitamins from the supermarket”), it was not possible to assess the amounts of vitamins and minerals consumed by participants. Dietary supplements were defined as any other supplement that could not be considered as a VMS, such as plant extracts not considered as phytotherapy by the Swiss compendium, cod liver oil, shark cartilage or amino acids. Vitamin-mineral and dietary supplement use was assessed for the first, second, and third follow-ups. Participants were categorized as consumers or non-consumers as described previously [22].

### Cognitive tests

The Mini-Mental State Examination (MMSE) was administered to subjects older than 65 years of age. The MMSE [23] has two sections, the first using verbal responses to assess orientation, memory, and attention, the second assessing the ability to name objects, to carry out verbal or written instruction, and to spontaneously write a sentence or reproduce a geometrical figure. Values range from 0 to 30. The test is used to screen cognitive deficiency, evaluate its intensity, and measure changes in states of confusion and dementia in older subjects. The MMSE has shown satisfactory concurrent validity with the Wechsler Intelligence test and the French translation was validated by Hoff [24].

Screening of cognitive impairment was conducted based on the Cognitive Complaint Questionnaire [25], the Grober and Buschke episodic memory test [26], the CERAD praxis items [27, 28], the lexical and semantic fluency tasks and the Stroop color test [29].

The Grober and Buschke test [26, 30] is used primarily to assess episodic memory by evaluating the ability to encode and retrieve information related to specific items through a list of 16 words from different semantic categories. The test comprises multiple phases: the learning and encoding phase, where participants are presented with the word list and later asked to recall independently as many words as possible; the immediate recall phase, which evaluates initial encoding effectiveness; this phase is followed by a distracting task to minimize short-term memory effects; the cued recall phase where participants receive cues to aid memory retrieval; the delayed recall phase where participants attempt free recall after a brief interval, followed by cued recall; finally, the recognition phase, they identify previously learned words from a larger set that includes distractor words. The phases of free recall and cued recall are repeated three times. Values for each test range from 0 to 16.

CERAD [27, 28] assesses multiple cognitive functions, including memory, attention, language, and executive functions, to identify potential cognitive deficits. The test consists of several subtests, such as verbal fluency, MMSE, Word List Learning, Recall and Recognition, and construction praxis. Values range from 0 to 11.

Stroop color test [29] involves the presentation of color words printed in incongruent ink colors (e.g., the word “green” displayed in red ink). Participants are required to identify the colour of the ink rather than read the word itself. Conversely, they are asked to read the word without considering the ink colour. These tasks assess their ability to suppress automatic reading responses associated with word reading or colour identification, thereby demonstrating their capacity to ignore distractions and manage conflicting information. Values range from 0 to 24.

### Other covariates

Smoking was self-reported and categorized as never, former (irrespective of the time since quitting smoking) and current. Education was categorized into high (university), middle (high school) and low (apprenticeship  or mandatory). Marital status was defined as living alone (single, divorced, widowed) or living with a partner. Alcohol consumption was self-reported in units (glasses of wine, glasses or bottles of beer, shots of spirits) and categorized as none, 1–13, 14–27 and 28 + units per week.

Dietary intake was assessed using a self-administered, semi-quantitative FFQ which also included portion size [31]. Briefly, this FFQ assesses the dietary intake of the previous 4 weeks and consists of 97 different food items. We computed a modified version of the Alternative Healthy Eating Index [32] by omitting the multivitamin supplement use component, to avoid overadjustment in the multivariable analyses.

Major depressive disorder (MDD) was assessed using the French version [33] of the semi-structured Diagnostic Interview for Genetic Studies (DIGS) [34]. Interviews were carried out by trained master-level psychologists. A senior psychologist reviewed all interviews and assessments. MDD was categorized as never, remitted, and current.

Blood pressure (BP) was measured using an Omron^®^ HEM-907 automated oscillometric sphygmomanometer after at least a 10-minute rest in a seated position, and the average of the last two measurements was used. Hypertension was defined by an SBP ≥ 140 mm Hg or a DBP ≥ 90 mm Hg or the presence of antihypertensive drug treatment.

Glucose was assessed by glucose dehydrogenase, with maximum inter and intra-batch CVs of 2.1% and 1.0%, respectively. Diabetes mellitus (DM) was defined as fasting plasma glucose ≥ 7.0 mmol/L and/or the presence of oral hypoglycaemic or insulin treatment.

### Inclusion and exclusion criteria

Participants were considered eligible if they were aged ≥ 65 years. Participants were excluded from the cross-sectional analyses if they missed (1) any cognitive test, or (2) any covariate.

### Statistical analysis

Statistical analysis was conducted using Stata version 18.0 for Windows (Stata Corp, College Station, TX, USA). For bivariate analysis, results were expressed as number of participants (percentage) for categorical variables and as average ± standard deviation or median [interquartile range] for continuous variables. Bivariate between-group comparisons were performed using the chi-square test for categorical variables and the student’s t-test or Kruskal-Wallis test. For multivariable analysis, results were expressed as adjusted mean ± standard error of the mean. Between-group comparisons were performed by analysis of variance adjusting for age (continuous), gender (man, woman), marital status (living alone, living in a couple), educational level (high, medium, low), hypertension (yes, no), diabetes (yes, no), alcohol consumption (none, 1–13, 14–27 and 28 + units per week), AHEI (continuous), and MDD (never, remitted, current).

A second analysis was conducted using the entire study period using a mixed model considering repeated measures and the evolution of responses with time. Two models were applied: the first only considered vitamin supplement use, and the second was adjusted as described previously.

A sensitivity analysis was conducted by considering vitamin and/or dietary supplement consumption. Statistical significance was considered for a two-sided test with *p* < 0.05.

## Results

### Characteristics of the sample

Of the initial 1434, 2033 and 1754 eligible participants from the first, second and third follow-ups, respectively, 925 (64.5%), 836 (41.1%), and 516 (29.4%) participants were included. The reasons for exclusion are indicated in Supplementary Fig. 1 and the characteristics of excluded and included participants are detailed in Supplementary Table 2. There were no consistent differences between included and excluded participants, except for education level, hypertension, and diabetes. In the second follow-up, participants with hypertension and lower levels of education were more likely to be excluded. Participants with diabetes and lower levels of education had a higher chance of being excluded from the third follow-up.

The characteristics of the included participants according to VMS consumption are indicated in supplementary Table 3. VMS consumers were more frequently women, lived less frequently in couple, presented less frequently with hypertension and with MDD (remitted or current).

### Association between vitamin and/or dietary supplement use and cognition

The bivariate and multivariable results of the cognition tests according to VMS consumption are shown in Table 1. The statistically significant differences observed in the bivariate analyses became nonsignificant after multivariate analyses. The exception was Stroop C in the multivariate analyses, where VMS consumers performed better than non-consumers at the third follow-up. When all study periods were considered, no differences were found between VMS consumers and non-consumers (Table 2). Similar results were obtained when dietary supplements were also considered (Supplementary Tables 4 and 5).

**Table 1:** Bivariate and multivariable analysis of the cognition status of the participants according to vitamin supplement consumption, for each study period, CoLaus|PsyCoLaus study, Lausanne, Switzerland

	2009–2013				2014–2018			
	No consumers	Consumers	Effect size	*P*-value	No consumers	Consumers	Effect size	*P*-value
**N**	650	275			502	334		
**MMSE**								
Median [IQR]	30 [29–30]	30 [29–30]		*† 0.524*	29 [29–30]	30 [29–30]		*† 0.037*
Average ± SEM	29.27 ± 0.06	29.28 ± 0.09	0.02 (-0.21; 0.24)	*0.892*	29.21 ± 0.06	29.28 ± 0.07	0.08 (-0.11; 0.26)	*0.432*
**Stroop C**								
Median [IQR]	24 [24–24]	24 [24–24]		*0.626*	24 [24–24]	24 [24–24]		*† 0.451*
Average ± SEM	23.93 ± 0.02	23.89 ± 0.04	-0.04 (-0.13; 0.04)	*0.304*	23.97 ± 0.01	23.96 ± 0.02	0 (-0.05; 0.04)	*0.852*
**Stroop W**								
Median [IQR]	24 [24–24]	24 [24–24]		*† 0.167*	24 [24–24]	24 [24–24]		*† 0.826*
Average ± SEM	23.95 ± 0.01	23.93 ± 0.02	-0.02 (-0.07; 0.03)	*0.352*	23.96 ± 0.01	23.96 ± 0.02	0 (-0.05; 0.04)	*0.860*
**Stroop CW**								
Median [IQR]	24 [23–24]	24 [23–24]		*† 0.301*	24 [23–24]	24 [23–24]		*† 0.727*
Average ± SEM	23.23 ± 0.07	23.27 ± 0.11	0.04 (-0.23; 0.31)	*0.774*	23.42 ± 0.07	23.35 ± 0.09	-0.07 (-0.30; 0.16)	*0.565*
**CERAD**								
Median [IQR]	11 [10–11]	11 [10–11]		*† 0.243*	10 [9–11]	10 [10–11]		*† 0.173*
Average ± SEM	10.46 ± 0.04	10.51 ± 0.06	0.05 (-0.10; 0.20)	*0.534*	10.07 ± 0.06	10.07 ± 0.08	0 (-0.21; 0.21)	*1.000*
	**2019–2021**							
	**No consumers**		**Consumers**		**Effect size**		**P-value**	
**N**	310		206					
**MMSE**								
Median [IQR]	30 [29–30]		30 [29–30]				*† 0.161*	
Average ± SEM	29.32 ± 0.08		29.21 ± 0.09		-0.10 (-0.36; 0.16)		*0.442*	
**Stroop C**								
Median [IQR]	24 [24–24]		24 [24–24]				*† 0.641*	
Average ± SEM	24.02 ± 0.08		23.72 ± 0.11		-0.30 (-0.58; -0.01)		*0.042*	
**Stroop W**								
Median [IQR]	24 [24–24]		24 [24–24]				*† 0.364*	
Average ± SEM	23.90 ± 0.03		23.99 ± 0.04		0.08 (-0.03; 0.20)		*0.166*	
**Stroop CW**								
Median [IQR]	24 [23–24]		24 [23–24]				*† 0.800*	
Average ± SEM	23.45 ± 0.09		23.39 ± 0.12		-0.06 (-0.38; 0.26)		*0.715*	
**CERAD**								
Median [IQR]	10 [10–11]		11 [10–11]				*† 0.005*	
Average ± SEM	10.09 ± 0.08		10.22 ± 0.10		0.13 (-0.15; 0.41)		*0.353*	

IQR, interquartile range; MMSE, mini-mental state examination; Stroop C, Stroop colour; Stroop W, Stroop Word. SD, standard deviation; SEM, standard error of the mean. For bivariate analysis, results are expressed as average ± standard deviation or median [interquartile range]. Between-group comparisons performed using student’s t-test or Kruskal-Wallis test (†). For multivariable analysis, results are expressed as adjusted mean ± standard error of the mean. Between-group comparisons performed by analysis of variance adjusting for age (continuous), gender (man, woman), marital status (living alone, living in couple), educational level (high, medium, low), hypertension (yes, no), diabetes (yes, no), alcohol consumption (none, 1–13, 14–27 and 28 + units per week), AHEI (continuous), and current major depression disorder (never, remitted, current)

**Table 2:** Bivariate and multivariable analysis of the cognition status of the participants according to vitamin supplement consumption, CoLaus|PsyCoLaus study, Lausanne, Switzerland, considering all study periods together

	No consumers	Consumers	Effect size	*P*-value
MMSE				
Bivariate	29.16 ± 0.04	29.25 ± 0.05	0.09 (-0.03; 0.21)	*0.127*
Multivariable	29.23 ± 0.04	29.26 ± 0.05	0.03 (-0.10; 0.17)	*0.616*
Stroop C				
Bivariate	23.94 ± 0.02	23.90 ± 0.02	-0.03 (-0.08; 0.02)	*0.246*
Multivariable	23.94 ± 0.02	23.88 ± 0.03	-0.06 (-0.12; 0)	*0.070*
Stroop W				
Bivariate	23.94 ± 0.01	23.94 ± 0.01	-0.01 (-0.04; 0.02)	*0.648*
Multivariable	23.95 ± 0.01	23.95 ± 0.01	0 (-0.04; 0.03)	*0.852*
Stroop CW				
Bivariate	23.23 ± 0.05	23.30 ± 0.06	0.08 (-0.08; 0.23)	*0.325*
Multivariable	23.30 ± 0.05	23.31 ± 0.07	0.01 (-0.15; 0.17)	
CERAD				
Bivariate	10.21 ± 0.03	10.27 ± 0.04	0.06 (-0.05; 0.16)	*0.300*
Multivariable	10.29 ± 0.03	10.30 ± 0.05	0.01 (-0.10; 0.13)	*0.843*

For bivariate and multivariable analysis, results are expressed as average ± standard error of the mean. Between-group comparisons performed by mixed model considering repeated measures and time trend. Multivariable analysis adjusting for age (continuous), gender (man, woman), marital status (living alone, living in couple), educational level (high, medium, low), hypertension (yes, no), diabetes (yes, no), alcohol consumption (none, 1–13, 14–27 and 28 + units per week), AHEI (continuous), and major depression disorder (never, remitted, current)

The bivariate and multivariable analysis of the cognition status with the Grober and Buschke test according to VMS consumption are shown in Table 3. Again, most significant associations observed in bivariate analyses were no longer significant after multivariable adjustment. In the first follow-up, VMS consumers showed statistically significantly higher scores in Free Recall 1 than non-consumers, while non-consumers obtained higher scores than consumers in Cued Recall 1. Similar findings regarding Free and Cued Recall 1 were obtained when all periods were considered (Table 4). Similar results were observed when dietary supplements were considered (Supplementary Tables 6 and 7).

**Table 3:** Bivariate and multivariable analysis of the cognition status (Grober and Buschke free and delayed recall test) of the participants according to vitamin supplement consumption, for each study period, CoLaus|PsyCoLaus study, Lausanne, Switzerland

	2009–2013				2014–2018			
	No consumers	Consumers	Effect size	*P*-value	No consumers	Consumers	Effect size	*P*-value
**N**	650	275			502	334		
**Identification**								
Median [IQR]	16 [16–16]	16 [16–16]		*† 0.126*	16 [16–16]	16 [16–16]		*† 1.000*
Average ± SEM	15.97 ± 0.01	15.98 ± 0.02	0.01 (-0.03; 0.05)	*0.581*	15.98 ± 0.03	15.93 ± 0.04	-0.05 (-0.15; 0.05)	*0.332*
**Immediate recall**								
Median [IQR]	16 [16–16]	16 [16–16]		*† 0.809*	16 [16–16]	16 [16–16]		*† 0.631*
Average ± SEM	15.73 ± 0.05	15.78 ± 0.08	0.05 (-0.15; 0.25)	*0.629*	15.94 ± 0.05	15.87 ± 0.07	-0.07 (-0.24; 0.11)	*0.439*
**Free recall 1**								
Median [IQR]	8 [7–10]	9 [8–11]		*† <0.001*	8 [7–10]	9 [7–11]		*† <0.001*
Average ± SEM	8.55 ± 0.10	9.11 ± 0.15	0.56 (0.2; 0.93)	*0.003*	8.51 ± 0.11	8.91 ± 0.14	0.40 (0.02; 0.78)	*0.040*
**Cued recall 1**								
Median [IQR]	7 [5–8]	6 [4–7]		*† <0.001*	7 [5–8]	6 [5–8]		*† 0.116*
Average ± SEM	6.48 ± 0.09	5.99 ± 0.14	-0.48 (-0.81; -0.16)	*0.004*	6.49 ± 0.11	6.28 ± 0.13	-0.21 (-0.56; 0.15)	*0.250*
**Free recall 2**								
Median [IQR]	10 [8–12]	11 [9–12]		*† 0.014*	10 [8–12]	11 [9–13]		*† 0.013*
Average ± SEM	10.10 ± 0.10	10.30 ± 0.16	0.20 (-0.18; 0.59)	*0.304*	10.20 ± 0.13	10.42 ± 0.16	0.22 (-0.21; 0.66)	*0.317*
**Cued recall 2**								
Median [IQR]	5 [4–7]	5 [3–6]		*† 0.008*	5 [4–7]	5 [3–7]		*† 0.095*
Average ± SEM	5.41 ± 0.09	5.11 ± 0.14	-0.30 (-0.64; 0.04)	*0.084*	5.19 ± 0.12	5.17 ± 0.14	-0.01 (-0.40; 0.37)	*0.947*
**Free recall 3**								
Median [IQR]	11 [9–13]	12 [10–13]		*† 0.040*	11 [9–13]	12 [10–13]		*† 0.004*
Average ± SEM	11.18 ± 0.10	11.31 ± 0.16	0.14 (-0.25; 0.52)	*0.485*	11.17 ± 0.13	11.41 ± 0.16	0.24 (-0.19; 0.66)	*0.274*
**Cued recall 3**								
Median [IQR]	4 [3–6]	4 [3–6]		*† 0.287*	4 [3–6]	4 [3–6]		*† 0.036*
Average ± SEM	4.55 ± 0.09	4.56 ± 0.14	0.01 (-0.32; 0.34)	*0.947*	4.46 ± 0.11	4.32 ± 0.14	-0.13 (-0.51; 0.24)	*0.489*
**Delayed free recall**								
Median [IQR]	12 [10–13]	12 [10–14]		*† 0.058*	12 [10–14]	12 [10–14]		*† 0.019*
Average ± SEM	11.49 ± 0.11	11.55 ± 0.17	0.06 (-0.34; 0.47)	*0.760*	11.94 ± 0.13	12.02 ± 0.16	0.07 (-0.36; 0.51)	*0.737*
**Delayed cued recall**								
Median [IQR]	4 [3–6]	4 [2–6]		*† 0.153*	4 [2–6]	4 [2–5]		*† 0.026*
Average ± SEM	4.32 ± 0.09	4.25 ± 0.15	-0.07 (-0.43; 0.28)	*0.683*	3.90 ± 0.12	3.87 ± 0.14	-0.03 (-0.41; 0.35)	*0.869*
**Recognition**								
Median [IQR]	48 [47–48]	48 [48–48]		*† 0.205*	48 [48–48]	48 [48–48]		*† 0.130*
Average ± SEM	44.95 ± 0.39	44.51 ± 0.62	-0.44 (-1.93; 1.05)	*0.560*	46.99 ± 0.32	46.05 ± 0.39	-0.95 (-1.99; 0.10)	*0.075*
	**2019–2021**							
	**No consumers**		**Consumers**		**Effect size**		**P-value**	
**N**	310		206					
**Identification**								
Median [IQR]	16 [16–16]		16 [16–16]				*† 0.382*	
Average ± SEM	15.99 ± 0.01		15.97 ± 0.02		-0.02 (-0.06; 0.03)		*0.455*	
**Immediate recall**								
Median [IQR]	16 [16–16]		16 [16–16]				*† 0.638*	
Average ± SEM	15.84 ± 0.10		15.79 ± 0.13		-0.05 (-0.39; 0.29)		*0.786*	
**Free recall 1**								
Median [IQR]	8 [6–10]		9 [7–11]				*† 0.003*	
Average ± SEM	8.63 ± 0.16		8.57 ± 0.20		-0.06 (-0.60; 0.48)		*0.830*	
**Cued recall 1**								
Median [IQR]	7 [5–8]		6 [4–8]				*† 0.018*	
Average ± SEM	6.38 ± 0.14		6.25 ± 0.19		-0.14 (-0.63; 0.36)		*0.593*	
**Free recall 2**								
Median [IQR]	10 [8–12]		11 [9–13]				*† 0.013*	
Average ± SEM	10.39 ± 0.16		10.15 ± 0.21		-0.24 (-0.79; 0.32)		*0.398*	
**Cued recall 2**								
Median [IQR]	5 [4–7]		5 [3–6]				*† 0.083*	
Average ± SEM	5.01 ± 0.14		5.20 ± 0.18		0.18 (-0.30; 0.66)		*0.463*	
**Free recall 3**								
Median [IQR]	11 [9–13]		12 [10–13]				*† 0.008*	
Average ± SEM	11.16 ± 0.16		11.17 ± 0.21		0.01 (-0.54; 0.57)		*0.964*	
**Cued recall 3**								
Median [IQR]	5 [3–6]		4 [3–6]				*† 0.017*	
Average ± SEM	4.55 ± 0.14		4.51 ± 0.19		-0.04 (-0.53; 0.46)		*0.881*	
**Delayed free recall**								
Median [IQR]	12 [10–14]		12 [11–14]				*† 0.039*	
Average ± SEM	12.08 ± 0.17		11.67 ± 0.22		-0.41 (-1; 0.19)		*0.179*	
**Delayed cued recall**								
Median [IQR]	4 [2–6]		4 [2–5]				*† 0.748*	
Average ± SEM	3.77 ± 0.15		4.22 ± 0.20		0.46 (-0.06; 0.98)		*0.082*	
**Recognition**								
Median [IQR]	48 [48–48]		48 [48–48]				*† 0.474*	
Average ± SEM	47.76 ± 0.05		47.78 ± 0.06		0.02 (-0.15; 0.18)		*0.848*	

IQR, interquartile range; MMSE, mini-mental state examination; SD, standard deviation; SEM, standard error of the mean. For bivariate analysis, results are expressed as average ± standard deviation or median [interquartile range]. Between-group comparisons performed using student’s t-test or Kruskal-Wallis test (†). For multivariable analysis, results are expressed as adjusted mean ± standard error of the mean. Between-group comparisons performed by analysis of variance adjusting for age (continuous), gender (man, woman), marital status (living alone, living in couple), educational level (high, medium, low), hypertension (yes, no), diabetes (yes, no), alcohol consumption (none, 1–13, 14–27 and 28 + units per week), AHEI (continuous), and current major depression disorder (never, remitted, current)

IQR, interquartile range; SD, standard deviation; SEM, standard error of the mean. For bivariate analysis, results are expressed as average ± standard deviation or median [interquartile range]. Between-group comparisons performed using student’s t-test or Kruskal-Wallis test (†). For multivariable analysis, results are expressed as adjusted mean ± standard error of the mean. Between-group comparisons performed by analysis of variance adjusting for age (continuous), gender (man, woman), marital status (living alone, living in couple), educational level (high, medium, low), hypertension (yes, no), diabetes (yes, no), alcohol consumption (none, 1–13, 14–27 and 28 + units per week), AHEI (continuous), and current major depression disorder (never, remitted, current)

**Table 4:** Bivariate and multivariable analysis of the cognition status (Grober and Buschke free and delayed recall test) of the participants according to vitamin supplement consumption, CoLaus|PsyCoLaus study, Lausanne, Switzerland, considering all study periods together

	No consumers	Consumers	Effect size	*P*-value
Identification				
Bivariate	15.96 ± 0.01	15.95 ± 0.02	-0.02 (-0.06; 0.03)	*0.513*
Multivariable	15.98 ± 0.01	15.96 ± 0.02	-0.02 (-0.06; 0.02)	*0.361*
**Immediate recall**				
Bivariate	15.82 ± 0.03	15.84 ± 0.04	0.02 (-0.08; 0.13)	*0.649*
Multivariable	15.82 ± 0.04	15.82 ± 0.05	0 (-0.12; 0.13)	*0.944*
**Free recall 1**				
Bivariate	8.43 ± 0.07	8.74 ± 0.09	0.55 (0.34; 0.76)	*< 0.001*
Multivariable	8.50 ± 0.07	8.88 ± 0.10	0.37 (0.13; 0.61)	*0.002*
**Cued recall 1**				
Bivariate	6.55 ± 0.06	6.16 ± 0.08	-0.39 (-0.58; -0.21)	*< 0.001*
Multivariable	6.49 ± 0.06	6.15 ± 0.09	-0.34 (-0.55; -0.12)	*0.003*
**Free recall 2**				
Bivariate	9.98 ± 0.08	10.29 ± 0.10	0.30 (0.07; 0.53)	*0.010*
Multivariable	10.13 ± 0.08	10.21 ± 0.11	0.08 (-0.18; 0.33)	*0.564*
**Cued recall 2**				
Bivariate	5.37 ± 0.06	5.10 ± 0.08	-0.27 (-0.47; -0.07)	*0.007*
Multivariable	5.29 ± 0.07	5.20 ± 0.09	-0.09 (-0.32; 0.14)	*0.442*
**Free recall 3**				
Bivariate	10.95 ± 0.08	11.34 ± 0.10	0.39 (0.16; 0.62)	*0.001*
Multivariable	11.10 ± 0.08	11.24 ± 0.10	0.14 (-0.11; 0.39)	*0.271*
**Cued recall 3**				
Bivariate	4.66 ± 0.06	4.40 ± 0.09	-0.27 (-0.46; -0.07)	*0.009*
Multivariable	4.57 ± 0.07	4.51 ± 0.09	-0.06 (-0.29; 0.16)	*0.578*
**Delayed free recall**				
Bivariate	11.53 ± 0.08	11.73 ± 0.10	0.21 (-0.03; 0.45)	*0.086*
Multivariable	11.66 ± 0.08	11.64 ± 0.11	-0.02 (-0.29; 0.24)	*0.878*
**Delayed cued recall**				
Bivariate	4.22 ± 0.07	4.05 ± 0.09	-0.17 (-0.38; 0.03)	*0.099*
Multivariable	4.13 ± 0.07	4.12 ± 0.10	-0.01 (-0.25; 0.22)	*0.914*
**Recognition**				
Bivariate	46.19 ± 0.19	46.17 ± 0.25	-0.01 (-0.61; 0.59)	*0.965*
Multivariable	46.19 ± 0.21	45.83 ± 0.30	-0.37 (-1.11; 0.38)	*0.337*

For bivariate and multivariable analysis, results are expressed as average ± standard error of the mean. Between-group comparisons performed by mixed model considering repeated measures and time trend. Multivariable analysis adjusting for age (continuous), gender (man, woman), marital status (living alone, living in couple), educational level (high, medium, low), hypertension (yes, no), diabetes (yes, no), alcohol consumption (none, 1–13, 14–27 and 28 + units per week), AHEI (continuous), and major depression disorder (never, remitted, current)

## Discussion

In this multiple cross-sectional study, no differences were found regarding cognition levels between VMS consumers and non-consumers. Similar findings were obtained when dietary supplements were considered.

### Association between vitamin and/or dietary supplement use and cognition

In this study, VMS consumers did not demonstrate a better cognitive performance when the MMSE test was used. Our findings are consistent with many multivitamin supplementation studies conducted in elderly people, be they cognitively healthy [15, 35] or presenting with mild cognitive impairment [14, 36, 37], which failed to find a significant effect of supplementation on cognition. Our results also agree with studies focusing on individual vitamin supplementation among elderly people, such as vitamin D [7, 38], or with studies assessing the association between blood vitamin B_12_ levels and cognition [39], which reported no change in cognitive tests.

Conversely, our findings differ from other studies, which reported significant improvements in cognition after supplementation with multivitamin [16], omega-3 fatty acids combined with other dietary supplements [17, 18], and vitamins B [4, 13, 40]. Still, the differences observed in some studies were relatively small and questionable regarding their clinical relevance: in the multivitamin study, the performance improved from a mean of 7.10 words at baseline to 7.81 words in the multivitamin group versus 7.21 words at baseline to 7.65 words after 1 year in the placebo group [16]. In a study of omega-3 fatty acids, the placebo group improved more than the intervention group in the Repeatable Battery for the Assessment of Neuropsychological Status (RBANS) immediate, and declined in two others [17]. One recent meta-analysis demonstrated the possible effectiveness of B and D vitamin supplementation on cognition among people with mild cognitive impairment [41]. Still, the studies included in this meta-analysis had small sample sizes and were conducted in people with cognitive disorders, which does not correspond to our sample.

One possible explanation for the result’s discrepancy may lie in the type of cognition tests applied. For instance, the MMSE and Montreal Cognitive Assessment (MoCA) are the two main generic cognitive tests used in the research examining the effect of VMS on cognitive function. Interestingly, the studies based on these tests often demonstrated greater benefits of VMS ingestion [8, 17, 40, 41], while the fewer studies using more specific cognition tests such as the Stroop Color and Word or CERAD [11, 12, 39] found no differences between VMS consumers and non-consumers. It’s important to note that MMSE and MoCA tests are intended to screen cognitive impairment rather than to conduct a comprehensive cognitive evaluation to ascertain the cognitive state [23, 42]. Hence, it is possible that the benefits from VMS supplementation reported by some studies might be due to the use of an inappropriate test to assess cognition.

VMS consumers performed better than non-consumers regarding the Stroop C test in the third follow-up, a finding also reported elsewhere [18]. Still, the difference observed was very small: 0.3 over 3 points as reported in another study [18] and can thus be considered as clinically negligible.

According to the Grober and Buschke tests, VMS consumers performed better in free recall, requiring fewer signals for providing a correct answer. Conversely, VMS non-consumers improved their scores with cueing. Because VMS studies do not use the Grober and Buschke tests, our results cannot be compared with those found in the literature. These differences, however, were small and devoid of clinical significance. The reasons for such differences are currently unknown and it would be of interest to replicate our study to confirm or infirm our findings.

Overall, our results suggest that VMS and/or dietary supplement consumption is not associated with improved cognition.

### Strengths and limitations

One of the study’s strengths is the sizable sample of apparently healthy, community-dwelling individuals. Additionally, a large battery of cognitive tests was administered, enabling testing of different facets of cognition. Lastly, the analyses were adjusted for the presence of a depressive episode at the time of the cognitive testing.

This work also has some limitations. First, it was not possible to characterize the content of vitamin supplements, as many participants were unable to specify the precise VMS they were taking. In addition, VMS vary considerably in composition, precluding the use of a generic component [21]. Second, the study was conducted in a single location, and results might not be generalizable to other settings. Third, only participants aged ≥ 65 years were included; hence, our results might not apply to younger participants [43], although the prevalence of cognitive decline is rare below 65 [20]. Fourthly, the number of participants aged 80 + was relatively small and was not representative of the corresponding population of canton Vaud. Finally, the cross-sectional design of this study restricts the ability to establish causal relationships or to observe changes in variables over time, which could be better assessed through longitudinal research.

### Implications for clinical practice

This study failed to find any association between vitamin or dietary supplements and cognitive performance in apparently healthy, community-dwelling participants, as shown by other randomized and epidemiological studies. Health practitioners should not prescribe such supplements as a preventive measure against cognitive decline in healthy elderly subjects devoid of vitamin or mineral deficiencies.

## Conclusion

In this cross-sectional population-based study, we found no clinically significant association between vitamin-mineral ± dietary supplement use and cognitive performance.

## Electronic supplementary material

Below is the link to the electronic supplementary material.

Supplementary Material 1

## Data Availability

The data of the CoLaus|PsyCoLaus study used in this article cannot be fully shared as they contain potentially sensitive personal information on participants. According to the Ethics Committee for Research of the Canton of Vaud, sharing these data would be a violation of the Swiss legislation concerning privacy protection. However, coded individual-level data that do not allow researchers to identify participants are available upon request to researchers who meet the criteria for data sharing of the CoLaus|PsyCoLaus Datacenter (CHUV, Lausanne, Switzerland). Any researcher affiliated with a public or private research institution who complies with the CoLaus|PsyCoLaus standards can submit a research application to research.colaus@chuv.ch or research.psycolaus@chuv.ch. Proposals requiring baseline data only will be evaluated by the baseline (local) Scientific Committee (SC) of the CoLaus and PsyCoLaus studies. Proposals requiring follow-up data will be evaluated by the follow-up (multicentric) SC of the CoLaus|PsyCoLaus cohort study. Detailed instructions for gaining access to the CoLaus|PsyCoLaus data used in this study are available at www.colaus-psycolaus.ch/professionals/how-to-collaborate/.
