# Supplementary material for: Vitamin-mineral supplements and cognition among adults aged 65 and older: multiple cross-sectional population-based studies
Source: Eur J Nutr. 2025 May 24;64(5):184. doi: 10.1007/s00394-025-03700-2 (PMC12103314; doi:10.1007/s00394-025-03700-2)
Supplement: Supplementary file 1 — Supplementary Material 1 [file 394_2025_3700_MOESM1_ESM.pdf]

Supplementary information for Vitamin-mineral supplements and cognition among adults aged 65 and older: multiple cross-sectional population-based studies

Daniela Marques, student [1]; Martin Preisig, MD, PhD [2]; Pedro-Marques Vidal, MD, PhD, FESC[3]

[1] University of Lausanne, Lausanne, Switzerland; [2] Department of Psychiatry, Lausanne University Hospital and University of Lausanne, Lausanne, Switzerland; [3] Department of Medicine, Internal Medicine, Lausanne University Hospital (CHUV) and University of Lausanne

Corresponding author:
Pedro Marques-Vidal
Office BH10-642
Department of Medicine, Internal Medicine
Lausanne University Hospital
Rue du Bugnon 46
1011 Lausanne
Switzerland
Phone: +41 (0)21 314 09 34
Fax: +41 (0)21 314 09 51
Email: Pedro-Manuel.Marques-Vidal@chuv.ch

Supplementary Figure 1: Flow of participants in the different survey periods, CoLaus|PsyCoLaus study, Lausanne, Switzerland.

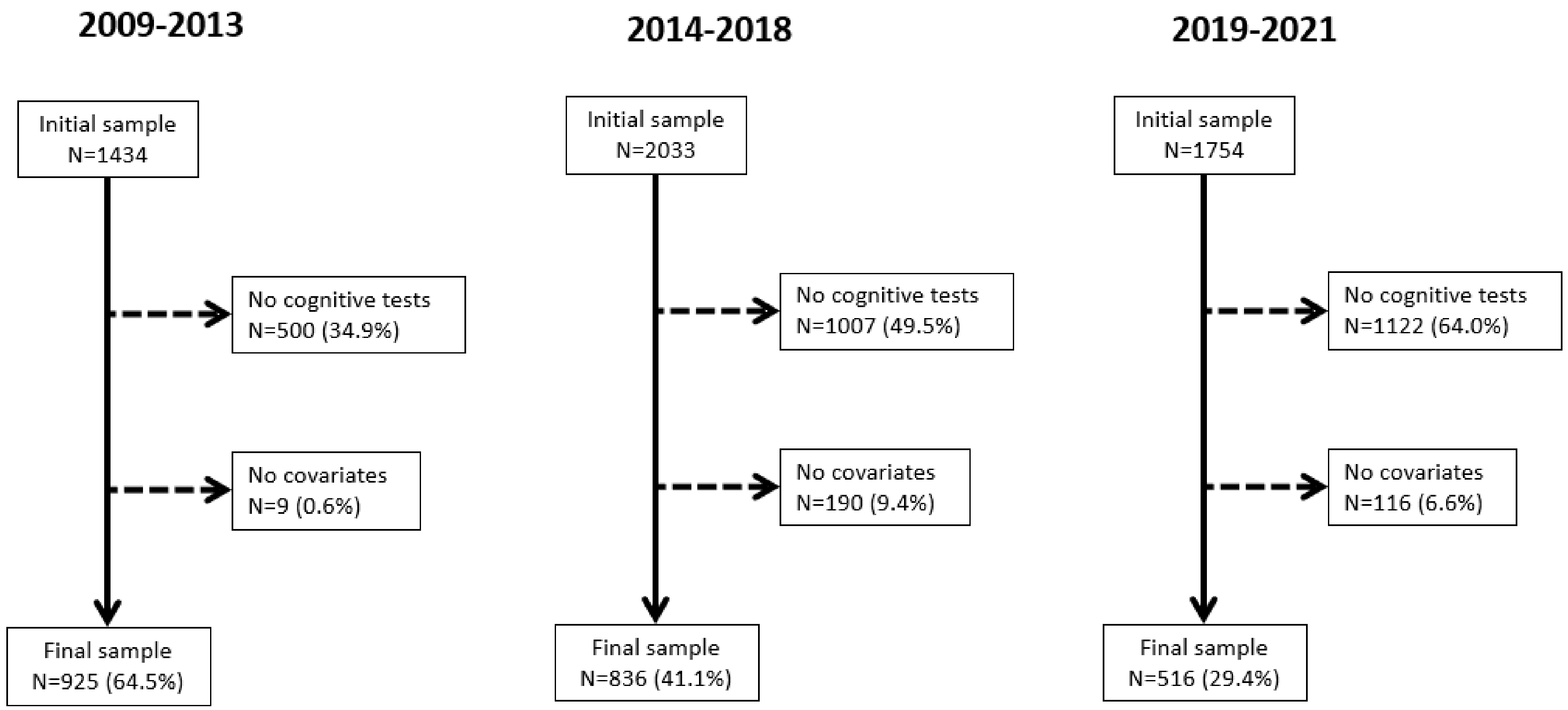

Supplementary table 1: comparison of age categories between the CoLaus|PsyCoLaus study and the corresponding population of canton Vaud, per study period.

	2009-2013		2014-2018		2019-2021	
	**Study**	**Canton**	**Study**	**Canton**	**Study**	**Canton**
**Overall**						
[65-70[	52.4%	43.4%	37.9%	39.5%	35.3%	36.0%
[70-75[	27.4%	30.9%	38.7%	35.8%	35.7%	34.7%
[75-80[	20.1%	25.7%	23.4%	24.7%	29.0%	29.2%
**Women**						
[65-70[	52.7%	42.1%	38.2%	38.9%	35.9%	37.3%
[70-75[	27.5%	31.0%	40.1%	35.5%	32.7%	34.3%
[75-80[	19.8%	26.8%	21.7%	25.6%	31.5%	28.5%
**Men**						
[65-70[	52.1%	45.0%	37.4%	40.2%	34.6%	35.0%
[70-75[	27.4%	30.8%	36.8%	36.1%	39.8%	35.1%
[75-80[	20.5%	24.2%	25.8%	23.7%	25.7%	29.9%

Results are expressed as percentage. Data was extracted from the statistics website of canton Vaud. https://www.vd.ch/etat-droit-finances/statistique/statistiques-par-domaine/01-population/etat-et-structure-de-la-population#c2058328, assessed 27 March 2025. For each study period of CoLaus|PsyCoLaus, data from the mid-point year was extracted (2011 for 2009-2013, 2016 for 2014-2018 and 2020 for 2019-2021).

Supplementary table 2: Characteristics of excluded and included participants in the different study periods, CoLaus|PsyCoLaus study, Lausanne, Switzerland.

	2009-2013			2014-2018			2019-2021		
	**Included**	**Excluded**	**P-value**	**Included**	**Excluded**	**P-value**	**Included**	**Excluded**	**P-value**
**N**	925	509		836	1197		516	1238	
**Women (%)**	534 (57.7)	271 (53.2)	*0.101*	491 (58.7)	712 (59.5)	*0.735*	290 (56.2)	775 (62.6)	*0.012*
**Age (years)**	70.9 ± 4.5	71.8 ± 4.6	*<0.001*	72.8 ± 5.2	74.0 ± 5.7	*<0.001*	73.7 ± 5.6	74.5 ± 6.2	*0.021*
**Education level (%)**			*0.078*			*0.007*			*0.001*
High	132 (14.3)	62 (12.2)		137 (16.4)	176 (14.7)		111 (21.5)	204 (16.5)	
Middle	244 (26.4)	114 (22.4)		232 (27.8)	271 (22.7)		152 (29.5)	303 (24.5)	
Low	549 (59.4)	333 (65.4)		467 (55.9)	749 (62.6)		253 (49.0)	731 (59.1)	
**Living in couple (%)**	509 (55.0)	285 (56.0)	*0.725*	497 (59.5)	515 (58.3)	*0.635*	305 (59.1)	568 (57.7)	*0.605*
**Smoking categories (%)**			*0.632*			*0.878*			*0.169*
Never	377 (40.8)	214 (43.3)		362 (43.3)	444 (44.3)		217 (42.1)	494 (46.2)	
Former	419 (45.3)	216 (43.7)		361 (43.2)	421 (42.0)		247 (47.9)	458 (42.8)	
Current	129 (14.0)	64 (13.0)		113 (13.5)	137 (13.7)		52 (10.1)	117 (10.9)	
**Alcohol intake (%)**			*0.022*			*<0.001*			*<0.001*
Nondrinkers	226 (24.4)	163 (32.0)		198 (25.5)	344 (37.9)		177 (34.3)	648 (52.3)	
1-13/week	547 (59.2)	268 (52.6)		464 (59.7)	440 (48.5)		272 (52.7)	477 (38.5)	
14-27/week	128 (13.8)	66 (13.0)		95 (12.2)	106 (11.7)		57 (11.1)	89 (7.2)	
28+/week	24 (2.6)	12 (2.4)		20 (2.6)	18 (2.0)		10 (1.9)	24 (1.9)	
**AHEI**	29.7 ± 10.2	29.6 ± 10.6	*0.796*	29.0 ± 10.3	28.6 ± 10.1	*0.507*	29.2 ± 10.3	28.5 ± 10.4	*0.223*
**Hypertension (%)**	632 (68.3)	364 (72.1)	*0.140*	516 (61.7)	807 (72.5)	*<0.001*	330 (64.0)	806 (68.1)	*0.097*
**Diabetes (%)**	173 (18.7)	97 (19.3)	*0.789*	105 (12.6)	211 (20.7)	*<0.001*	59 (11.4)	178 (15.6)	*0.024*

AHEI, alternative healthy eating index. Results are expressed as number of participants (column percentage) for categorical variables and as average ± standard deviation for continuous variables. Between-group comparisons performed using chi-square test for categorical variables and student's t-test for continuous variables.

Supplementary Table 3: characteristics of the participants according to vitamin/mineral supplement consumption, for each study period, CoLaus|PsyCoLaus study, Lausanne, Switzerland.

	2009-2013			2014-2018			2019-2021		
	**No**	**Yes**	**P-value**	**No**	**Yes**	**P-value**	**No**	**Yes**	**P-value**
**N**	636	287		455	365		289	221	
**Women (%)**	308 (48.4)	224 (78.1)	*<0.001*	199 (43.7)	282 (77.3)	*<0.001*	118 (40.8)	166 (75.1)	*<0.001*
**Age (years)**	70.8 ± 4.5	71.3 ± 4.5	*0.109*	72.4 ± 5.1	73.2 ± 5.2	*0.040*	73.3 ± 5.5	74.3 ± 5.7	*0.051*
**Education level (%)**			*0.258*			*0.716*			*0.434*
High	92 (14.5)	40 (13.9)		72 (15.8)	64 (17.5)		68 (23.5)	42 (19.0)	
Middle	158 (24.8)	86 (30)		123 (27.0)	102 (28.0)		81 (28.0)	69 (31.2)	
Low	386 (60.7)	161 (56.1)		260 (57.1)	199 (54.5)		140 (48.4)	110 (49.8)	
**Living in couple (%)**	372 (58.5)	135 (47.0)	*0.001*	302 (66.4)	186 (51.0)	*<0.001*	183 (63.3)	121 (54.8)	*0.051*
**Smoking status (%)**			*0.635*			*0.279*			*0.018*
Never	253 (39.8)	123 (42.9)		190 (41.8)	166 (45.5)		123 (42.6)	89 (40.3)	
Former	291 (45.8)	127 (44.3)		195 (42.9)	156 (42.7)		128 (44.3)	118 (53.4)	
Current	92 (14.5)	37 (12.9)		70 (15.4)	43 (11.8)		38 (13.2)	14 (6.3)	
**Alcohol intake (%)**			*0.047*			*0.007*			*0.113*
Nondrinkers	145 (22.3)	81 (29.5)		105 (22.5)	93 (29.9)		104 (33.5)	73 (35.4)	
1-13/week	388 (59.7)	159 (57.8)		277 (59.4)	187 (60.1)		158 (51.0)	114 (55.4)	
14-27/week	97 (14.9)	31 (11.3)		69 (14.8)	26 (8.4)		39 (12.6)	18 (8.7)	
28+/week	20 (3.1)	4 (1.5)		15 (3.2)	5 (1.6)		9 (2.9)	1 (0.5)	
**AHEI**	31.2 ± 9.8	26.4 ± 10.4	*<0.001*	31.3 ± 10.5	25.7 ± 9.1	*<0.001*	31.6 ± 10.1	25.6 ± 9.6	*<0.001*
**Hypertension (%)**	456 (71.7)	175 (61.0)	*0.001*	294 (64.6)	210 (57.5)	*0.038*	199 (68.9)	129 (58.4)	*0.014*
**Diabetes (%)**	128 (20.1)	44 (15.3)	*0.083*	64 (14.1)	40 (11.0)	*0.184*	38 (13.2)	21 (9.5)	*0.202*
**MDD (%)**			*0.522*			*0.002*			*0.059*
Never	424 (67.7)	180 (64.0)		294 (64.8)	193 (52.9)		168 (60.4)	112 (54.4)	
Remitted	171 (27.3)	87 (31.0)		147 (32.4)	154 (42.2)		102 (36.7)	79 (38.4)	
Current	31 (5.0)	14 (5.0)		13 (2.9)	18 (4.9)		8 (2.9)	15 (7.3)	

AHEI, alternative healthy eating index; MDD; major depression disorder. Results are expressed as number of participants (column percentage) for categorical variables and as average ± standard deviation for continuous variables. Between-group comparisons performed using the chi-square test for categorical variables and the student's t-test for continuous variables.

Supplementary table 4: bivariate and multivariable analysis of the cognition status of the participants according to vitamin-mineral or dietary supplement consumption, for each study period, CoLaus|PsyCoLaus study, Lausanne, Switzerland.

	**2009-2013**				**2014-2018**			
	**No**	**Yes**	**Effect size**	**P-value**	**No**	**Yes**	**Effect size**	**P-value**
**N**	636	287			455	365		
**MMSE**								
Median [IQR]	30 [29 - 30]	30 [29 - 30]		*† 0.434*	29 [29 - 30]	30 [29 - 30]		*† 0.034*
Average ± SEM	29.27 ± 0.06	29.27 ± 0.09	0 (-0.22 ; 0.23)	*0.983*	29.2 ± 0.06	29.29 ± 0.07	0.09 (-0.10 ; 0.27)	*0.360*
**Stroop C**								
Median [IQR]	24 [24 - 24]	24 [24 - 24]		*† 0.898*	24 [24 - 24]	24 [24 - 24]		*† 0.331*
Average ± SEM	23.94 ± 0.02	23.88 ± 0.03	-0.06 (-0.14; 0.02)	*0.156*	23.97 ± 0.01	23.97 ± 0.02	0 (-0.04 ; 0.04)	*0.961*
**Stroop W**								
Median [IQR]	24 [24 - 24]	24 [24 - 24]		*† 0.059*	24 [24 - 24]	24 [24 - 24]		*† 0.440*
Average ± SEM	23.96 ± 0.01	23.93 ± 0.02	-0.03 (-0.08; 0.02)	*0.223*	23.96 ± 0.01	23.96 ± 0.02	0.01 (-0.04 ; 0.05)	*0.768*
**Stroop CW**								
Median [IQR]	24 [23 - 24]	24 [23 - 24]		*† 0.278*	24 [23 - 24]	24 [23 - 24]		*† 0.962*
Average ± SEM	23.23 ± 0.07	23.26 ± 0.11	0.03 (-0.24 ; 0.29)	*0.837*	23.41 ± 0.07	23.36 ± 0.08	-0.05 (-0.28; 0.18)	*0.668*
**CERAD**								
Median [IQR]	11 [10 - 11]	11 [10 - 11]		*† 0.149*	10 [9 - 11]	10 [10 - 11]		*† 0.083*
Average ± SEM	10.46 ± 0.04	10.53 ± 0.06	0.07 (-0.08 ; 0.22)	*0.345*	10.03 ± 0.07	10.11 ± 0.07	0.08 (-0.13; 0.28)	*0.454*

IQR, interquartile range; MMSE, mini-mental state examination; SD, standard deviation; SEM, standard error of the mean. For bivariate analysis, results are expressed as average ± standard deviation or median [interquartile range]. Between-group comparisons performed using student's t-test or Kruskal-Wallis test (†). For multivariable analysis, results are expressed as adjusted mean ± standard error of the mean. Between-group comparisons performed by analysis of variance adjusting for age (continuous), gender (man, woman), marital status (living alone, living in couple), educational level (high, medium, low), hypertension (yes, no), diabetes (yes, no), alcohol consumption (none, 1-13, 14-27 and 28+ units per week), AHEI (continuous), and current major depression disorder (never, remitted, current).

Supplementary table 4 (continued): bivariate and multivariable analysis of the cognition status of the participants according to vitamin-mineral or dietary supplement consumption, for each study period, CoLaus|PsyCoLaus study, Lausanne, Switzerland.

	2019-2021			
	**No**	**Yes**	**Effect size**	**P-value**
**N**	289	221		
**MMSE**				
Median [IQR]	30 [29 - 30]	30 [29 - 30]		*† 0.081*
Average ± SEM	29.28 ± 0.08	29.25 ± 0.09	-0.03 (-0.28 ; 0.22)	*0.815*
**Stroop C**				
Median [IQR]	24 [24 - 24]	24 [24 - 24]		*† 0.835*
Average ± SEM	24.02 ± 0.09	23.75 ± 0.10	-0.27 (-0.55 ; 0.01)	*0.059*
**Stroop W**				
Median [IQR]	24 [24 - 24]	24 [24 - 24]		*† 0.235*
Average ± SEM	23.90 ± 0.04	23.99 ± 0.04	0.09 (-0.02 ; 0.21)	*0.123*
**Stroop CW**				
Median [IQR]	24 [23 - 24]	24 [23 - 24]		*† 0.665*
Average ± SEM	23.48 ± 0.1	23.38 ± 0.11	-0.1 (-0.41 ; 0.21)	*0.532*
**CERAD**				
Median [IQR]	10 [9 - 11]	11 [10 - 11]		*† 0.003*
Average ± SEM	10.08 ± 0.08	10.23 ± 0.10	0.15 (-0.12 ; 0.42)	*0.274*

IQR, interquartile range; MMSE, mini-mental state examination; SD, standard deviation; SEM, standard error of the mean. For bivariate analysis, results are expressed as average ± standard deviation or median [interquartile range]. Between-group comparisons performed using student's t-test or Kruskal-Wallis test (†). For multivariable analysis, results are expressed as adjusted mean ± standard error of the mean. Between-group comparisons performed by analysis of variance adjusting for age (continuous), gender (man, woman), marital status (living alone, living in couple), educational level (high, medium, low), hypertension (yes, no), diabetes (yes, no), alcohol consumption (none, 1-13, 14-27 and 28+ units per week), AHEI (continuous), and current major depression disorder (never, remitted, current).

Supplementary table 5: bivariate and multivariable analysis of the cognition status of the participants according to vitamin-mineral or dietary supplement consumption, CoLaus|PsyCoLaus study, Lausanne, Switzerland, considering all study periods together.

	**No consumers**	**Consumers**	**Effect size**	**P-value**
MMSE				
Bivariate	29.15 ± 0.04	29.26 ± 0.05	0.10 (-0.01 ; 0.22)	*0.080*
Multivariable	29.23 ± 0.04	29.27 ± 0.05	0.05 (-0.08 ; 0.18)	*0.477*
Stroop C				
Bivariate	23.94 ± 0.02	23.91 ± 0.02	-0.03 (-0.08 ; 0.02)	*0.199*
Multivariable	23.95 ± 0.02	23.89 ± 0.03	-0.06 (-0.12 ; 0)	*0.056*
Stroop W				
Bivariate	23.94 ± 0.01	23.94 ± 0.01	0 (-0.04 ; 0.03)	*0.773*
Multivariable	23.95 ± 0.01	23.95 ± 0.01	0 (-0.04 ; 0.03)	*0.925*
Stroop CW				
Bivariate	23.22 ± 0.05	23.31 ± 0.06	0.08 (-0.07 ; 0.23)	*0.277*
Multivariable	23.31 ± 0.05	23.31 ± 0.06	0 (-0.16 ; 0.16)	*0.989*
CERAD				
Bivariate	10.22 ± 0.03	10.27 ± 0.04	0.05 (-0.05 ; 0.16)	*0.321*
Multivariable	10.29 ± 0.03	10.32 ± 0.05	0.03 (-0.08 ; 0.15)	*0.568*

For bivariate and multivariable analysis, results are expressed as average ± standard error of the mean. Between-group comparisons performed by mixed model considering repeated measures and time trend. Multivariable analysis adjusting for age (continuous), gender (man, woman), marital status (living alone, living in couple), educational level (high, medium, low), hypertension (yes, no), diabetes (yes, no), alcohol consumption (none, 1-13, 14-27 and 28+ units per week), AHEI (continuous), and major depression disorder (never, remitted, current).

Supplementary table 6: bivariate and multivariable analysis of the cognition status (Buschke tests) of the participants according to vitamin-mineral or dietary supplement consumption, for each study period, CoLaus|PsyCoLaus study, Lausanne, Switzerland

	**2009-2013**				**2014-2018**			
	**No consumers**	**Consumers**	**Effect size**	**P-value**	**No consumers**	**Consumers**	**Effect size**	**P-value**
N	636	287			455	365		
**Identification**								
Median [IQR]	16 [16 - 16]	16 [16 - 16]		*† 0.103*	16 [16 - 16]	16 [16 - 16]		*† 0.778*
Average ± SEM	15.97 ± 0.01	15.98 ± 0.02	0.01 (-0.03 ; 0.05)	*0.513*	15.98 ± 0.03	15.94 ± 0.04	-0.04 (-0.14 ; 0.06)	*0.434*
**Immediate recall**								
Median [IQR]	16 [16 - 16]	16 [16 - 16]		*† 0.809*	16 [16 - 16]	16 [16 - 16]		*† 0.576*
Average ± SEM	15.73 ± 0.05	15.79 ± 0.08	0.06 (-0.14 ; 0.25)	*0.573*	15.93 ± 0.06	15.88 ± 0.06	-0.04 (-0.22 ; 0.13)	*0.610*
**Free recall 1**								
Median [IQR]	8 [7 - 10]	9 [8 - 11]		*†<0.001*	8 [7 - 10]	9 [7 - 11]		*†<0.001*
Average ± SEM	8.55 ± 0.10	9.09 ± 0.15	0.53 (0.17 ; 0.90)	*0.004*	8.51 ± 0.12	8.87 ± 0.13	0.36 (-0.01 ; 0.73)	*0.059*
**Cued recall 1**								
Median [IQR]	7 [5 - 8]	6 [4 - 7]		*†<0.001*	7 [5 - 8]	6 [5 - 8]		*† 0.110*
Average ± SEM	6.47 ± 0.09	6.02 ± 0.13	-0.45 (-0.77; -0.12)	*0.007*	6.49 ± 0.11	6.33 ± 0.13	-0.16 (-0.51 ; 0.19)	*0.372*
**Free recall 2**								
Median [IQR]	10 [8 - 12]	10 [9 - 12]		*† 0.023*	10 [8 - 12]	10 [9 - 12]		*† 0.056*
Average ± SEM	10.11 ± 0.10	10.29 ± 0.16	0.18 (-0.20 ; 0.56)	*0.354*	10.23 ± 0.14	10.37 ± 0.16	0.13 (-0.30 ; 0.56)	*0.551*
**Cued recall 2**								
Median [IQR]	5 [4 - 7]	5 [4 - 7]		*† 0.019*	5 [4 - 7]	5 [3 - 7]		*† 0.219*
Average ± SEM	5.40 ± 0.09	5.14 ± 0.14	-0.25 (-0.59 ; 0.08)	*0.139*	5.16 ± 0.12	5.21 ± 0.14	0.05 (-0.33 ; 0.43)	*0.809*
**Free recall 3**								
Median [IQR]	11 [9 - 13]	12 [10 - 13]		*† 0.064*	11 [9 - 13]	12 [10 - 13]		*† 0.016*
Average ± SEM	11.19 ± 0.10	11.30 ± 0.15	0.11 (-0.27 ; 0.48)	*0.571*	11.20 ± 0.14	11.38 ± 0.15	0.17 (-0.24 ; 0.59)	*0.417*
**Cued recall 3**								
Median [IQR]	4 [3 - 6]	4 [3 - 6]		*† 0.347*	4 [3 - 6]	4 [3 - 6]		*† 0.126*
Average ± SEM	4.54 ± 0.09	4.56 ± 0.13	0.02 (-0.31 ; 0.34)	*0.925*	4.42 ± 0.12	4.36 ± 0.13	-0.06 (-0.43 ; 0.31)	*0.761*
**Delayed free recall**								
Median [IQR]	12 [10 - 13]	12 [10 - 14]		*† 0.039*	12 [10 - 14]	12 [10 - 14]		*† 0.047*
Average ± SEM	11.48 ± 0.11	11.58 ± 0.16	0.11 (-0.29 ; 0.51)	*0.594*	11.97 ± 0.14	11.98 ± 0.15	0.01 (-0.42 ; 0.44)	*0.965*
**Delayed cued recall**								

Median [IQR]	4 [3 - 6]	4 [2 - 6]		*† 0.092*	4 [2 - 5]	4 [2 - 5]		*† 0.147*
Average ± SEM	4.35 ± 0.10	4.21 ± 0.14	-0.14 (-0.49 ; 0.21)	*0.432*	3.84 ± 0.12	3.95 ± 0.13	0.11 (-0.27 ; 0.48)	*0.570*
**Recognition**								
Median [IQR]	48 [47 - 48]	48 [48 - 48]		*† 0.166*	48 [48 - 48]	48 [48 - 48]		*† 0.107*
Average ± SEM	44.99 ± 0.40	44.42 ± 0.60	-0.57 (-2.03 ; 0.90)	*0.448*	46.88 ± 0.34	46.23 ± 0.37	-0.66 (-1.70 ; 0.38)	*0.213*

IQR, interquartile range; SD, standard deviation; SEM, standard error of the mean. For bivariate analysis, results are expressed as average ± standard deviation or median [interquartile range]. Between-group comparisons performed using student's t-test or Kruskal-Wallis test (†). For multivariable analysis, results are expressed as adjusted mean ± standard error of the mean. Between-group comparisons performed by analysis of variance adjusting for age (continuous), gender (man, woman), marital status (living alone, living in couple), educational level (high, medium, low), hypertension (yes, no), diabetes (yes, no), alcohol consumption (none, 1-13, 14-27 and 28+ units per week), AHEI (continuous), and major depression disorder (never, remitted, current).

Supplementary table 6 (continued): bivariate and multivariable analysis of the cognition status (Buschke tests) of the participants according to vitamin-mineral or dietary supplement consumption, for each study period, CoLaus|PsyCoLaus study, Lausanne, Switzerland

	2019-2021			
	**No consumers**	**Consumers**	**Effect size**	**P-value**
N	289	221		
**Identification**				
Median [IQR]	16 [16 - 16]	16 [16 - 16]		*† 0.736*
Average ± SEM	15.99 ± 0.01	15.96 ± 0.02	-0.02 (-0.07 ; 0.02)	*0.306*
**Immediate recall**				
Median [IQR]	16 [16 - 16]	16 [16 - 16]		*† 0.919*
Average ± SEM	15.86 ± 0.10	15.76 ± 0.12	-0.1 (-0.44 ; 0.23)	*0.547*
**Free recall 1**				
Median [IQR]	8 [6 - 10]	9 [7 - 11]		*† 0.002*
Average ± SEM	8.56 ± 0.16	8.62 ± 0.19	0.06 (-0.46 ; 0.58)	*0.822*
**Cued recall 1**				
Median [IQR]	7 [5 - 8]	6 [4 - 8]		*† 0.009*
Average ± SEM	6.45 ± 0.15	6.22 ± 0.18	-0.23 (-0.72 ; 0.26)	*0.351*
**Free recall 2**				
Median [IQR]	10 [8 - 12]	11 [9 - 13]		*† 0.008*
Average ± SEM	10.3 ± 0.17	10.21 ± 0.20	-0.09 (-0.63 ; 0.45)	*0.743*
**Cued recall 2**				
Median [IQR]	5 [4 - 7]	5 [3 - 6]		*† 0.042*
Average ± SEM	5.10 ± 0.15	5.12 ± 0.17	0.03 (-0.45 ; 0.50)	*0.916*
**Free recall 3**				
Median [IQR]	11 [9 - 13]	12 [10 - 13]		*† 0.010*
Average ± SEM	11.12 ± 0.17	11.17 ± 0.2	0.05 (-0.49 ; 0.59)	*0.858*
**Cued recall 3**				
Median [IQR]	5 [3 - 6]	4 [3 - 6]		*† 0.032*
Average ± SEM	4.55 ± 0.15	4.56 ± 0.18	0.01 (-0.48 ; 0.49)	*0.976*
**Delayed free recall**				
Median [IQR]	12 [10 - 14]	12 [11 - 14]		*† 0.022*
Average ± SEM	11.99 ± 0.18	11.76 ± 0.21	-0.24 (-0.81 ; 0.34)	*0.425*
**Delayed cued recall**				
Median [IQR]	4 [2 - 6]	4 [2 - 5]		*† 0.666*
Average ± SEM	3.88 ± 0.16	4.08 ± 0.19	0.2 (-0.31 ; 0.71)	*0.434*
**Recognition**				
Median [IQR]	48 [48 - 48]	48 [48 - 48]		*† 0.523*
Average ± SEM	47.75 ± 0.05	47.78 ± 0.06	0.03 (-0.13 ; 0.19)	*0.691*

IQR, interquartile range; SD, standard deviation; SEM, standard error of the mean. For bivariate analysis, results are expressed as average ± standard deviation or median [interquartile range]. Between-group comparisons performed using student's t-test or Kruskal-Wallis test (†). For multivariable analysis, results are expressed as adjusted mean ± standard error of the mean. Between-group comparisons performed by analysis of variance adjusting for age (continuous), gender (man, woman), marital status (living alone, living in couple), educational level (high, medium, low), hypertension (yes, no), diabetes (yes, no), alcohol consumption (none, 1-13, 14-27 and 28+ units per week), AHEI (continuous), and major depression disorder (never, remitted, current).

Supplementary table 7: bivariate and multivariable analysis of the cognition status (Buschke tests) of the participants according to vitamin-mineral or dietary supplement consumption, CoLaus|PsyCoLaus study, Lausanne, Switzerland, considering all study periods together.

	No consumers	Consumers	Effect size	P-value
**Identification**				
Bivariate	15.96 ± 0.01	15.95 ± 0.02	-0.01 (-0.06 ; 0.03)	*0.613*
Multivariable	15.97 ± 0.01	15.96 ± 0.02	-0.02 (-0.06 ; 0.02)	*0.431*
**Immediate recall**				
Bivariate	15.81 ± 0.03	15.85 ± 0.04	0.03 (-0.07 ; 0.13)	*0.555*
Multivariable	15.81 ± 0.04	15.82 ± 0.05	0.01 (-0.11 ; 0.14)	*0.828*
**Free recall 1**				
Bivariate	8.36 ± 0.07	8.89 ± 0.09	0.53 (0.32 ; 0.74)	*<0.001*
Multivariable	8.50 ± 0.07	8.85 ± 0.09	0.36 (0.12 ; 0.59)	*0.003*
**Cued recall 1**				
Bivariate	6.56 ± 0.06	6.17 ± 0.08	-0.4 (-0.58 ; -0.21)	*<0.001*
Multivariable	6.49 ± 0.07	6.18 ± 0.08	-0.31 (-0.53 ; -0.1)	*0.004*
**Free recall 2**				
Bivariate	9.98 ± 0.08	10.26 ± 0.10	0.28 (0.05 ; 0.5)	*0.017*
Multivariable	10.13 ± 0.08	10.2 ± 0.10	0.07 (-0.18 ; 0.32)	*0.583*
**Cued recall 2**				
Bivariate	5.38 ± 0.07	5.12 ± 0.08	-0.26 (-0.45 ; -0.06)	*0.010*
Multivariable	5.29 ± 0.07	5.21 ± 0.09	-0.08 (-0.30 ; 0.14)	*0.478*
**Free recall 3**				
Bivariate	10.96 ± 0.08	11.31 ± 0.10	0.35 (0.12 ; 0.58)	*0.003*
Multivariable	11.11 ± 0.08	11.23 ± 0.10	0.12 (-0.12 ; 0.37)	*0.317*
**Cued recall 3**				
Bivariate	4.66 ± 0.07	4.42 ± 0.08	-0.23 (-0.43 ; -0.04)	*0.020*
Multivariable	4.56 ± 0.07	4.53 ± 0.09	-0.03 (-0.25 ; 0.19)	*0.797*
**Delayed free recall**				
Bivariate	11.51 ± 0.08	11.74 ± 0.10	0.23 (-0.01 ; 0.46)	*0.057*
Multivariable	11.64 ± 0.08	11.69 ± 0.10	0.05 (-0.21 ; 0.31)	*0.701*
**Delayed cued recall**				
Bivariate	4.23 ± 0.07	4.05 ± 0.08	-0.19 (-0.39 ; 0.02)	*0.071*
Multivariable	4.16 ± 0.07	4.10 ± 0.09	-0.06 (-0.29 ; 0.17)	*0.589*
**Recognition**				
Bivariate	46.14 ± 0.19	46.21 ± 0.24	0.08 (-0.52 ; 0.67)	*0.804*
Multivariable	46.13 ± 0.22	45.90 ± 0.28	-0.24 (-0.97 ; 0.50)	*0.529*

For bivariate and multivariable analysis, results are expressed as average ± standard error of the mean. Between-group comparisons performed by mixed model considering repeated measures and time trend. Multivariable analysis adjusting for age (continuous), gender (man, woman), marital status (living alone, living in couple), educational level (high, medium, low), hypertension (yes, no), diabetes (yes, no), alcohol consumption (none, 1-13, 14-27 and 28+ units per week), AHEI (continuous), and major depression disorder (never, remitted, current).
